# *N*′-(2-Hy­droxy­benzyl­idene)-3-methyl­benzohydrazide

**DOI:** 10.1107/S1600536811049944

**Published:** 2011-11-25

**Authors:** Zeng-Xin Liu

**Affiliations:** a Experimental Center, Linyi University, Linyi 276005, People’s Republic of China

## Abstract

The title compound, C_15_H_14_N_2_O_2_, is the product of the reaction of 2-hy­droxy­benzaldehyde and 3-methyl­benzo­hydrazide. The dihedral angle between the substituted benzene rings is 19.5 (3)° and an intra­molecular O—H⋯N hydrogen bond generates an *S*(6) ring motif. In the crystal, mol­ecules are linked by N—H⋯O hydrogen bonds to generate *C*(4) chains propagating in [001] and C—H⋯O inter­actions to the same O-atom acceptor reinforce the chains.

## Related literature

For reference bond lengths, see: Allen *et al.* (1987 ▶). For related structues, see: Horkaew *et al.* (2011 ▶); Fun *et al.* (2011 ▶); Su *et al.* (2011 ▶); Hashemian *et al.* (2011 ▶); Promdet *et al.* (2011 ▶).
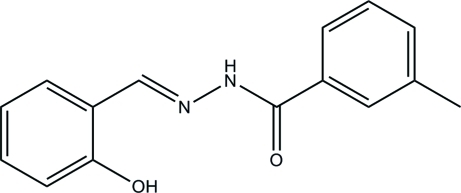


## Experimental

### 

#### Crystal data

C_15_H_14_N_2_O_2_
*M* *_r_* = 254.28
Monoclinic, 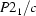

*a* = 11.042 (2) Å
*b* = 13.588 (3) Å
*c* = 8.7936 (15) Å
β = 94.406 (2)°
*V* = 1315.5 (4) Å^3^
*Z* = 4
Mo *K*α radiation
μ = 0.09 mm^−1^
*T* = 298 K
0.17 × 0.17 × 0.15 mm

#### Data collection

Bruker SMART 1K CCD diffractometer
Absorption correction: multi-scan (*SADABS*; Sheldrick, 1996 ▶) *T*_min_ = 0.985, *T*_max_ = 0.987
9633 measured reflections
2686 independent reflections
1528 reflections with *I* > 2σ(*I*)
*R*_int_ = 0.052

#### Refinement

*R*[*F* ^2^ > 2σ(*F* ^2^)] = 0.062
*wR*(*F* ^2^) = 0.186
*S* = 1.04
2686 reflections
177 parameters
1 restraint
H atoms treated by a mixture of independent and constrained refinement
Δρ_max_ = 0.59 e Å^−3^
Δρ_min_ = −0.24 e Å^−3^

### 

Data collection: *SMART* (Bruker, 2007 ▶); cell refinement: *SAINT* (Bruker, 2007 ▶); data reduction: *SAINT*; program(s) used to solve structure: *SHELXS97* (Sheldrick, 2008 ▶); program(s) used to refine structure: *SHELXL97* (Sheldrick, 2008 ▶); molecular graphics: *SHELXTL* (Sheldrick, 2008 ▶); software used to prepare material for publication: *SHELXL97*.

## Supplementary Material

Crystal structure: contains datablock(s) I, global. DOI: 10.1107/S1600536811049944/hb6528sup1.cif

Structure factors: contains datablock(s) I. DOI: 10.1107/S1600536811049944/hb6528Isup2.hkl

Supplementary material file. DOI: 10.1107/S1600536811049944/hb6528Isup3.cml

Additional supplementary materials:  crystallographic information; 3D view; checkCIF report

## Figures and Tables

**Table 1:** Hydrogen-bond geometry (Å, °)

*D*—H⋯*A*	*D*—H	H⋯*A*	*D*⋯*A*	*D*—H⋯*A*
O1—H1⋯N1	0.82	1.91	2.624 (2)	146
N2—H2⋯O2^i^	0.90 (1)	1.91 (1)	2.793 (3)	168 (3)
C7—H7⋯O2^i^	0.93	2.49	3.229 (2)	137

Symmetry code: (i) 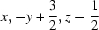.

**Fig. 1.**
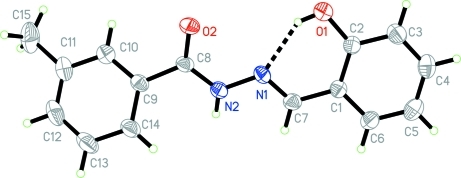
The molecular structure of the title compound, showing 30% probability displacement ellipsoids. The intramolecular O—H···N hydrogen bond is shown as a dashed line.

**Fig. 2.**
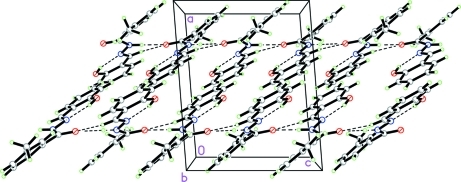
The molecular packing of the title compound, showing the N—H···O, O—H···N, and C—H···O hydrogen-bonds (dashed lines).

## supplementary crystallographic information

### Comment

Recently, the compounds derived from the condensation reaction of carbonyl-containing compounds with substituted benzohydrazides have received considerable attention. In this paper, the title new compound, derived from the reaction of 2-hydroxybenzaldehyde with 3-methylbenzohydrazide, is reported.

The molecule of the compound, Fig. 1, displays a *trans*-configuration about the C7 ═N1 bond. The torsion angle of C7—N1—N2—C8 is 7.0 (3)°. The dihedral angle between the C1—C6 and C9—C14 benzene rings is 19.5 (3)°, indicating the molecule of the compound is twisted. Overall, the bond distances are within normal values (Allen *et al.*, 1987), and are comparable with those reported in similar compounds (Horkaew *et al.*, 2011; Fun *et al.*, 2011; Su *et al.*, 2011; Hashemian *et al.*, 2011; Promdet *et al.*, 2011). In the crystal, molecules are linked by N—H···O intermolecular hydrogen bonds (Table 1) to form one-dimensional chains along the *c* axis (Fig. 2).

### Experimental

The title compound was synthesized by the reaction of 2-hydroxybenzaldehyde (1 mmol, 0.12 g) with 4-methylbenzohydrazide (1 mmol, 0.15 g) in absolute methanol (30 ml) at ambient condition. Colorless prism-shaped single crystals were obtained by slow evaporation of the solution at room temperature after several days.

### Refinement

The amide H atom was located in a difference map and was refined isotropically, with N—H = 0.90 (1) Å. The remaining H atoms were positioned geometrically and allowed to ride on their parent atoms, with C—H = 0.93 Å for aromatic and CH and 0.96 Å for CH_3_ atoms, and with O—H = 0.82 Å. The *U*_iso_(H) values were constrained to be 1.5*U*_eq_ of C15 and O1 atoms, and 1.2*U*_eq_ for the remaining C atoms. A rotating group model was used for the methyl group.

### Crystal data

**Table:**

C_15_H_14_N_2_O_2_	*F*(000) = 536
*M**_r_* = 254.28	*D*_x_ = 1.284 Mg m^−^^3^
Monoclinic, *P*2_1_/*c*	Mo *K*α radiation, λ = 0.71073 Å
*a* = 11.042 (2) Å	Cell parameters from 1290 reflections
*b* = 13.588 (3) Å	θ = 2.4–24.5°
*c* = 8.7936 (15) Å	µ = 0.09 mm^−^^1^
β = 94.406 (2)°	*T* = 298 K
*V* = 1315.5 (4) Å^3^	Prism, colorless
*Z* = 4	0.17 × 0.17 × 0.15 mm

### Data collection

**Table:**

Bruker SMART 1K CCD diffractometer	2686 independent reflections
Radiation source: fine-focus sealed tube	1528 reflections with *I* > 2σ(*I*)
graphite	*R*_int_ = 0.052
ω scan	θ_max_ = 26.5°, θ_min_ = 2.4°
Absorption correction: multi-scan (*SADABS*; Sheldrick, 1996)	*h* = −13→13
*T*_min_ = 0.985, *T*_max_ = 0.987	*k* = −17→15
9633 measured reflections	*l* = −10→10

### Refinement

**Table:**

Refinement on *F*^2^	Primary atom site location: structure-invariant direct methods
Least-squares matrix: full	Secondary atom site location: difference Fourier map
*R*[*F*^2^ > 2σ(*F*^2^)] = 0.062	Hydrogen site location: inferred from neighbouring sites
*wR*(*F*^2^) = 0.186	H atoms treated by a mixture of independent and constrained refinement
*S* = 1.04	*w* = 1/[σ^2^(*F*_o_^2^) + (0.0872*P*)^2^ + 0.1389*P*] where *P* = (*F*_o_^2^ + 2*F*_c_^2^)/3
2686 reflections	(Δ/σ)_max_ < 0.001
177 parameters	Δρ_max_ = 0.59 e Å^−^^3^
1 restraint	Δρ_min_ = −0.24 e Å^−^^3^

### Special details

**Table:**

Geometry. All e.s.d.'s (except the e.s.d. in the dihedral angle between two l.s. planes) are estimated using the full covariance matrix. The cell e.s.d.'s are taken into account individually in the estimation of e.s.d.'s in distances, angles and torsion angles; correlations between e.s.d.'s in cell parameters are only used when they are defined by crystal symmetry. An approximate (isotropic) treatment of cell e.s.d.'s is used for estimating e.s.d.'s involving l.s. planes.
Refinement. Refinement of *F*^2^ against ALL reflections. The weighted *R*-factor *wR* and goodness of fit *S* are based on *F*^2^, conventional *R*-factors *R* are based on *F*, with *F* set to zero for negative *F*^2^. The threshold expression of *F*^2^ > σ(*F*^2^) is used only for calculating *R*-factors(gt) *etc*. and is not relevant to the choice of reflections for refinement. *R*-factors based on *F*^2^ are statistically about twice as large as those based on *F*, and *R*- factors based on ALL data will be even larger.

### Fractional atomic coordinates and isotropic or equivalent isotropic displacement parameters (Å^2^)

**Table:**

	*x*	*y*	*z*	*U*_iso_*/*U*_eq_	
N1	0.28488 (18)	0.65695 (15)	0.0597 (2)	0.0486 (5)	
N2	0.22818 (19)	0.72924 (16)	−0.0306 (2)	0.0521 (6)	
O1	0.42021 (18)	0.59365 (14)	0.2981 (2)	0.0661 (6)	
H1	0.3787	0.6335	0.2473	0.099*	
O2	0.22190 (17)	0.83426 (13)	0.16687 (18)	0.0610 (6)	
C1	0.3578 (2)	0.49285 (18)	0.0816 (3)	0.0463 (6)	
C2	0.4170 (2)	0.50626 (19)	0.2260 (3)	0.0488 (6)	
C3	0.4770 (2)	0.4277 (2)	0.2997 (3)	0.0645 (8)	
H3	0.5176	0.4367	0.3953	0.077*	
C4	0.4765 (3)	0.3370 (2)	0.2314 (4)	0.0701 (9)	
H4	0.5163	0.2846	0.2818	0.084*	
C5	0.4180 (3)	0.3224 (2)	0.0897 (4)	0.0696 (8)	
H5	0.4176	0.2605	0.0445	0.083*	
C6	0.3600 (2)	0.39998 (19)	0.0152 (3)	0.0601 (7)	
H6	0.3214	0.3903	−0.0814	0.072*	
C7	0.2970 (2)	0.57306 (19)	−0.0011 (3)	0.0508 (6)	
H7	0.2660	0.5630	−0.1013	0.061*	
C8	0.2014 (2)	0.81577 (18)	0.0313 (3)	0.0457 (6)	
C9	0.1440 (2)	0.89021 (19)	−0.0753 (3)	0.0471 (6)	
C10	0.1681 (2)	0.98853 (19)	−0.0468 (3)	0.0549 (7)	
H10	0.2194	1.0056	0.0379	0.066*	
C11	0.1186 (2)	1.0620 (2)	−0.1397 (3)	0.0634 (8)	
C12	0.0402 (3)	1.0358 (3)	−0.2601 (3)	0.0768 (10)	
H12	0.0048	1.0844	−0.3233	0.092*	
C13	0.0125 (3)	0.9383 (3)	−0.2897 (3)	0.0796 (10)	
H13	−0.0418	0.9219	−0.3719	0.095*	
C14	0.0652 (2)	0.8645 (2)	−0.1976 (3)	0.0617 (8)	
H14	0.0476	0.7987	−0.2183	0.074*	
C15	0.1510 (3)	1.1678 (2)	−0.1097 (5)	0.1026 (13)	
H15A	0.1779	1.1969	−0.2006	0.154*	
H15B	0.2148	1.1717	−0.0294	0.154*	
H15C	0.0809	1.2025	−0.0798	0.154*	
H2	0.226 (3)	0.718 (2)	−0.1311 (13)	0.080*	

### Atomic displacement parameters (Å^2^)

**Table:**

	*U*^11^	*U*^22^	*U*^33^	*U*^12^	*U*^13^	*U*^23^
N1	0.0553 (12)	0.0510 (13)	0.0390 (12)	0.0021 (10)	0.0003 (9)	0.0082 (10)
N2	0.0706 (14)	0.0517 (13)	0.0332 (11)	0.0040 (11)	−0.0023 (10)	0.0029 (10)
O1	0.0816 (14)	0.0590 (13)	0.0556 (12)	0.0063 (10)	−0.0080 (10)	−0.0021 (10)
O2	0.0881 (13)	0.0607 (12)	0.0324 (10)	0.0018 (9)	−0.0064 (9)	−0.0003 (8)
C1	0.0501 (14)	0.0469 (15)	0.0427 (15)	−0.0014 (11)	0.0093 (11)	0.0031 (11)
C2	0.0543 (14)	0.0473 (16)	0.0455 (15)	0.0020 (12)	0.0085 (11)	0.0005 (12)
C3	0.0666 (18)	0.069 (2)	0.0576 (18)	0.0114 (15)	0.0015 (13)	0.0035 (15)
C4	0.0721 (19)	0.0573 (19)	0.082 (2)	0.0160 (15)	0.0138 (17)	0.0101 (17)
C5	0.0748 (19)	0.0540 (18)	0.082 (2)	0.0016 (15)	0.0173 (17)	−0.0099 (16)
C6	0.0688 (18)	0.0511 (17)	0.0603 (18)	−0.0028 (13)	0.0039 (14)	−0.0046 (14)
C7	0.0592 (15)	0.0535 (17)	0.0396 (14)	−0.0043 (12)	0.0029 (11)	−0.0007 (13)
C8	0.0514 (14)	0.0506 (16)	0.0345 (14)	−0.0042 (11)	−0.0006 (11)	0.0025 (12)
C9	0.0482 (14)	0.0589 (17)	0.0344 (13)	0.0035 (11)	0.0038 (10)	0.0039 (12)
C10	0.0514 (14)	0.0579 (17)	0.0555 (17)	0.0001 (12)	0.0045 (12)	0.0047 (14)
C11	0.0569 (16)	0.0629 (19)	0.072 (2)	0.0131 (14)	0.0162 (15)	0.0207 (15)
C12	0.081 (2)	0.090 (3)	0.060 (2)	0.0348 (19)	0.0108 (16)	0.0262 (18)
C13	0.075 (2)	0.111 (3)	0.0500 (18)	0.031 (2)	−0.0128 (14)	−0.0029 (18)
C14	0.0644 (17)	0.075 (2)	0.0441 (16)	0.0152 (14)	−0.0061 (13)	−0.0042 (14)
C15	0.083 (2)	0.064 (2)	0.161 (4)	0.0066 (18)	0.016 (2)	0.038 (2)

### Geometric parameters (Å, °)

**Table:**

N1—C7	1.270 (3)	C6—H6	0.9300
N1—N2	1.382 (3)	C7—H7	0.9300
N2—C8	1.338 (3)	C8—C9	1.487 (3)
N2—H2	0.895 (10)	C9—C14	1.375 (3)
O1—C2	1.345 (3)	C9—C10	1.381 (4)
O1—H1	0.8200	C10—C11	1.376 (4)
O2—C8	1.223 (3)	C10—H10	0.9300
C1—C6	1.392 (3)	C11—C12	1.362 (4)
C1—C2	1.395 (3)	C11—C15	1.500 (4)
C1—C7	1.447 (3)	C12—C13	1.380 (4)
C2—C3	1.390 (4)	C12—H12	0.9300
C3—C4	1.371 (4)	C13—C14	1.389 (4)
C3—H3	0.9300	C13—H13	0.9300
C4—C5	1.374 (4)	C14—H14	0.9300
C4—H4	0.9300	C15—H15A	0.9600
C5—C6	1.373 (4)	C15—H15B	0.9600
C5—H5	0.9300	C15—H15C	0.9600
			
C7—N1—N2	117.0 (2)	O2—C8—C9	120.8 (2)
C8—N2—N1	119.7 (2)	N2—C8—C9	116.0 (2)
C8—N2—H2	124.0 (19)	C14—C9—C10	119.3 (2)
N1—N2—H2	115.1 (19)	C14—C9—C8	122.2 (2)
C2—O1—H1	109.5	C10—C9—C8	118.5 (2)
C6—C1—C2	118.6 (2)	C11—C10—C9	122.1 (3)
C6—C1—C7	119.6 (2)	C11—C10—H10	118.9
C2—C1—C7	121.8 (2)	C9—C10—H10	118.9
O1—C2—C3	117.6 (2)	C12—C11—C10	118.1 (3)
O1—C2—C1	122.5 (2)	C12—C11—C15	121.0 (3)
C3—C2—C1	119.8 (2)	C10—C11—C15	120.9 (3)
C4—C3—C2	120.0 (3)	C11—C12—C13	121.1 (3)
C4—C3—H3	120.0	C11—C12—H12	119.5
C2—C3—H3	120.0	C13—C12—H12	119.5
C3—C4—C5	120.9 (3)	C12—C13—C14	120.4 (3)
C3—C4—H4	119.6	C12—C13—H13	119.8
C5—C4—H4	119.6	C14—C13—H13	119.8
C6—C5—C4	119.4 (3)	C9—C14—C13	118.9 (3)
C6—C5—H5	120.3	C9—C14—H14	120.5
C4—C5—H5	120.3	C13—C14—H14	120.5
C5—C6—C1	121.3 (3)	C11—C15—H15A	109.5
C5—C6—H6	119.4	C11—C15—H15B	109.5
C1—C6—H6	119.4	H15A—C15—H15B	109.5
N1—C7—C1	121.6 (2)	C11—C15—H15C	109.5
N1—C7—H7	119.2	H15A—C15—H15C	109.5
C1—C7—H7	119.2	H15B—C15—H15C	109.5
O2—C8—N2	123.1 (2)		

### Hydrogen-bond geometry (Å, °)

**Table:**

*D*—H···*A*	*D*—H	H···*A*	*D*···*A*	*D*—H···*A*
O1—H1···N1	0.82	1.91	2.624 (2)	146
N2—H2···O2^i^	0.90 (1)	1.91 (1)	2.793 (3)	168 (3)
C7—H7···O2^i^	0.93	2.49	3.229 (2)	137

Symmetry codes: (i) *x*, −*y*+3/2, *z*−1/2.
